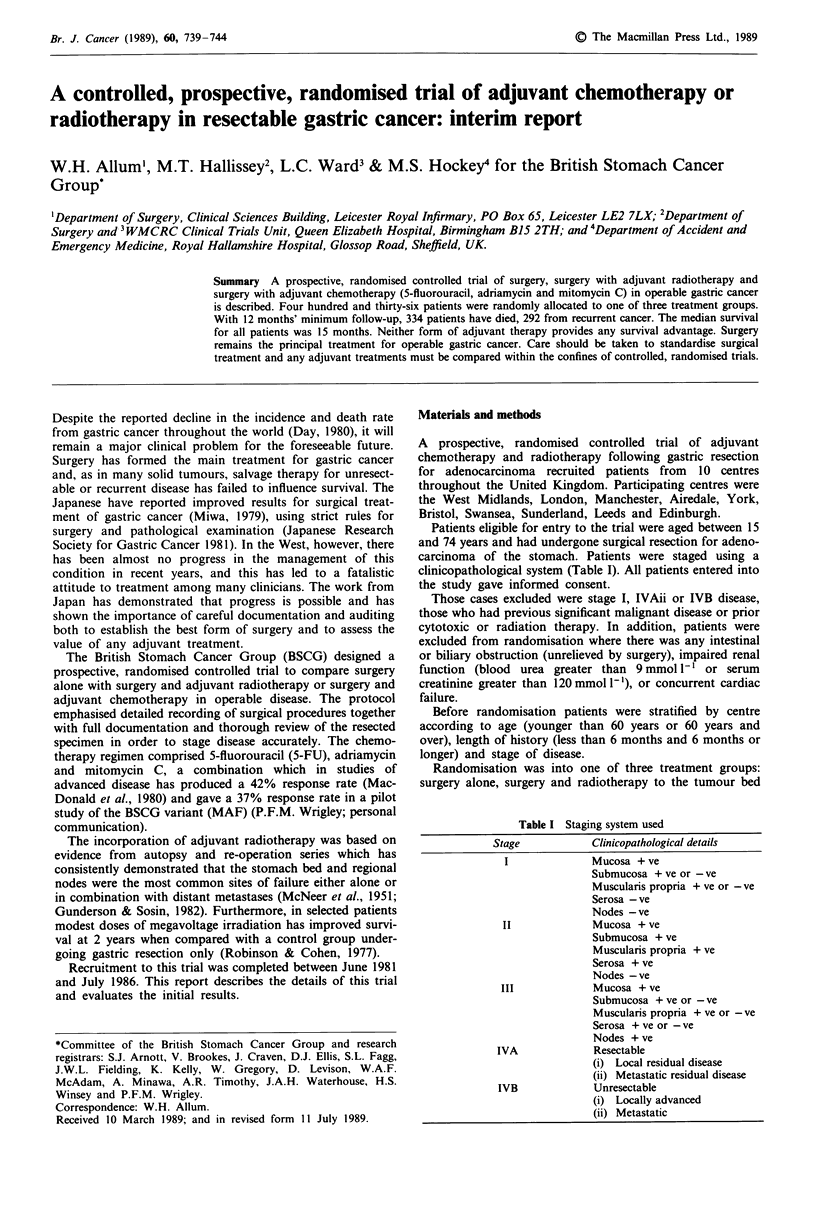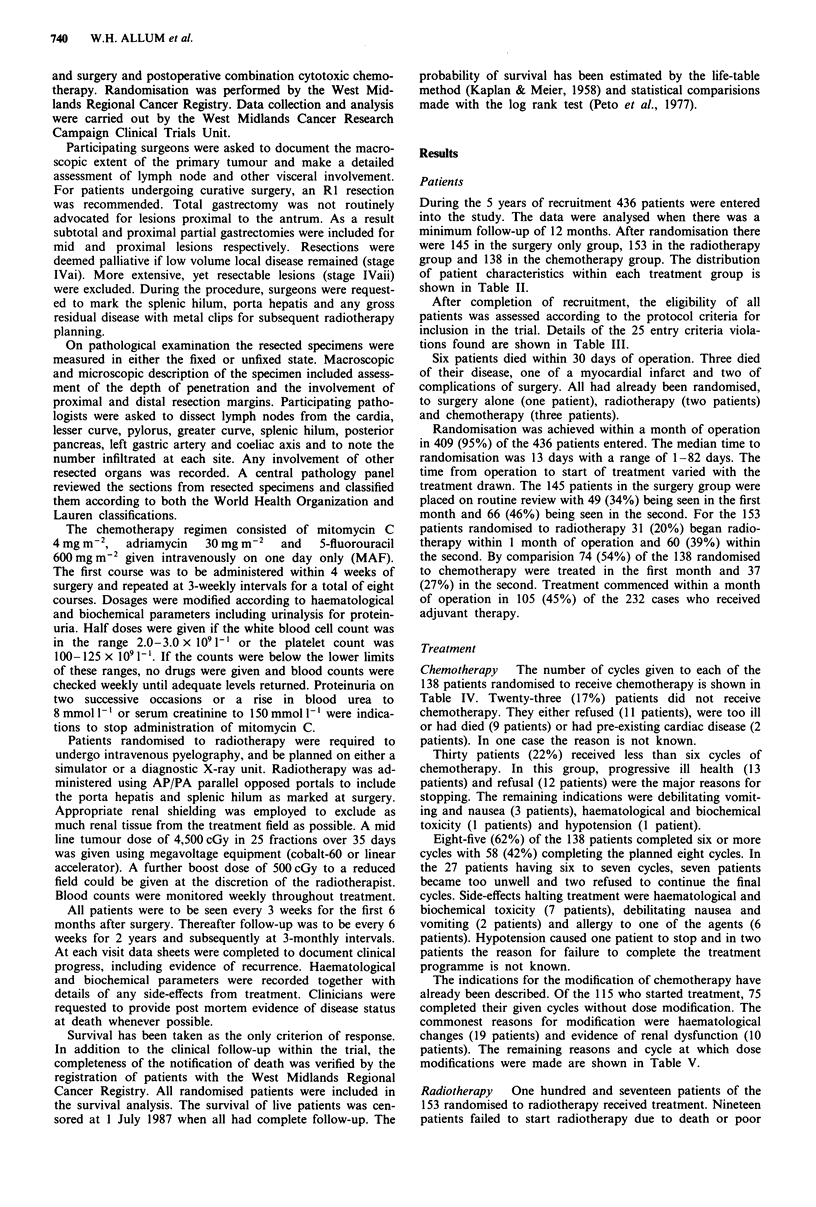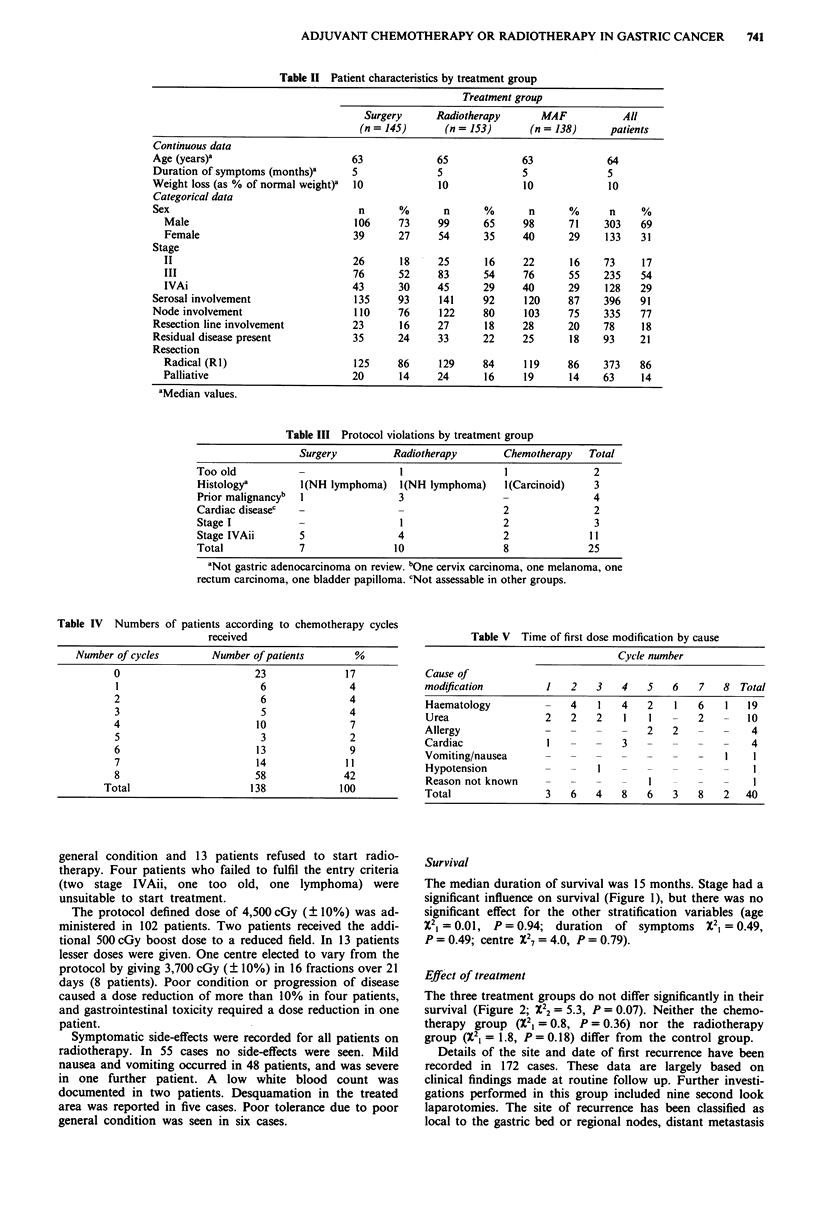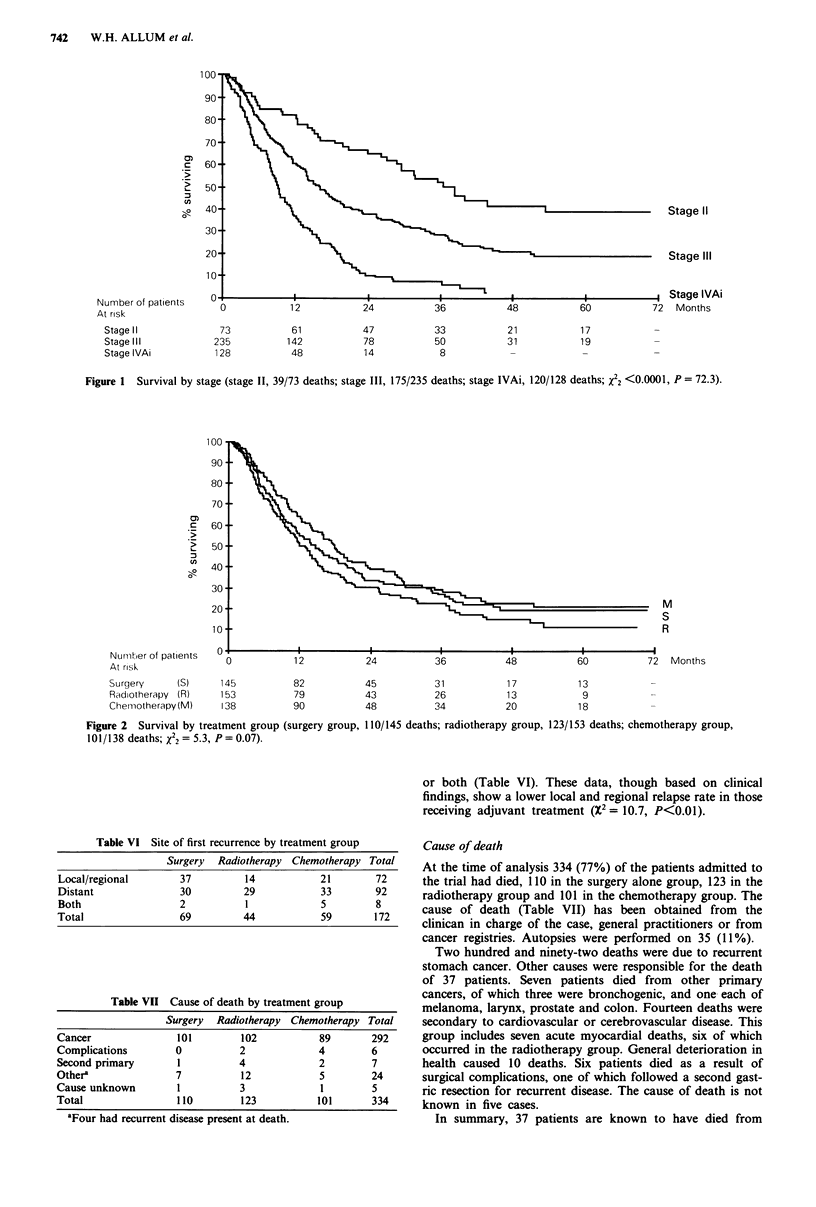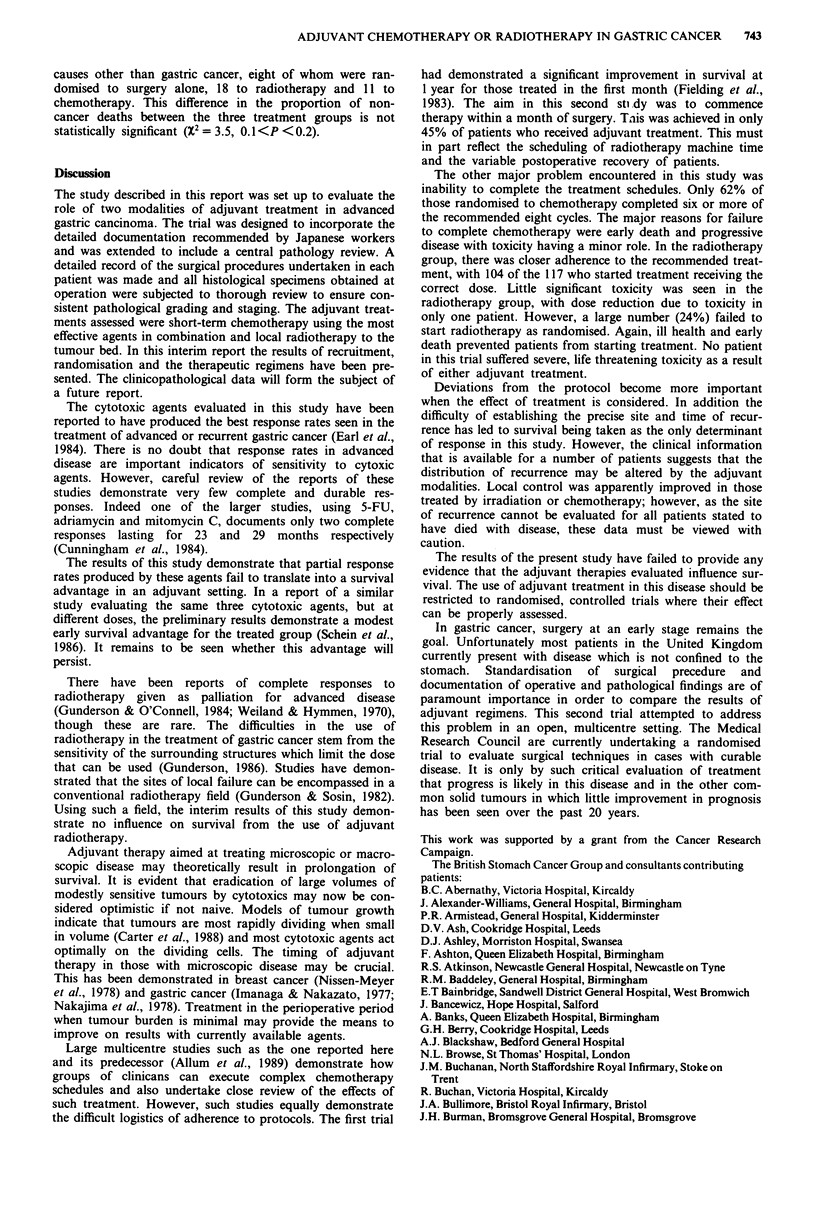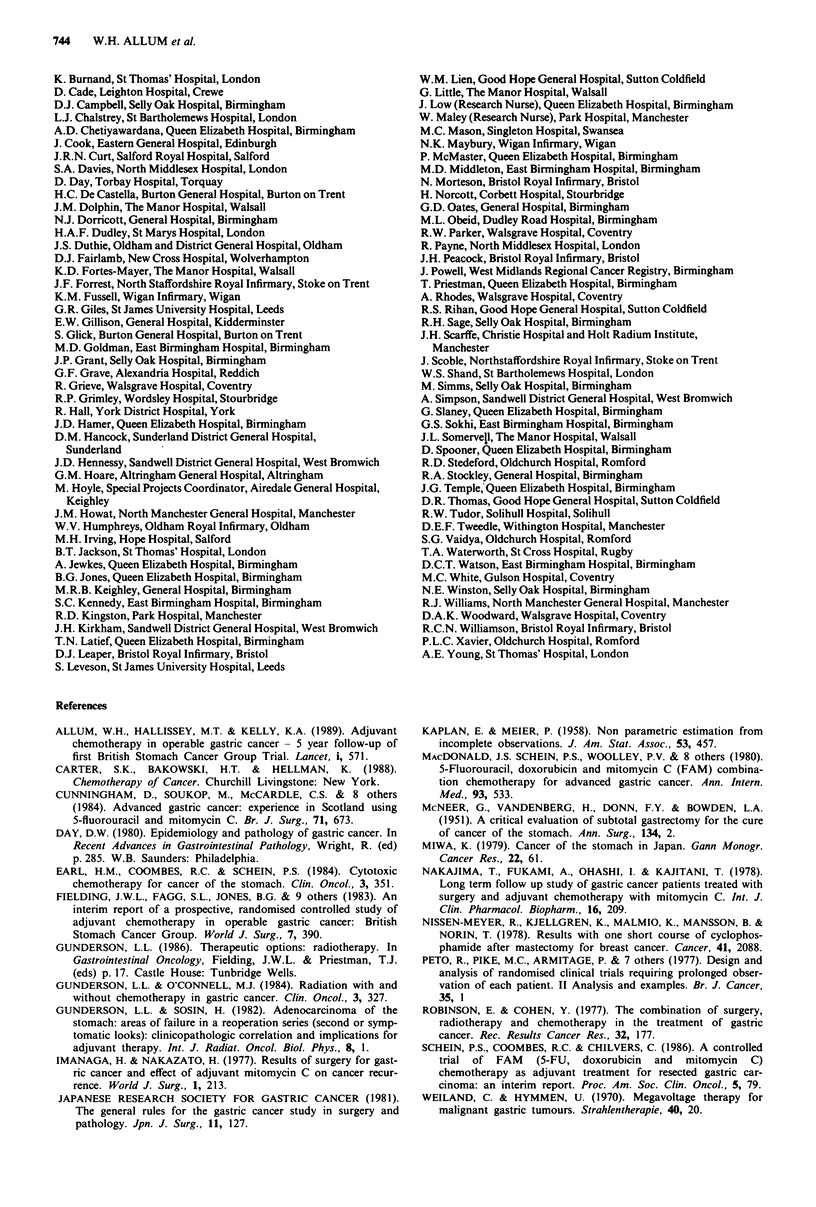# A controlled, prospective, randomised trial of adjuvant chemotherapy or radiotherapy in resectable gastric cancer: interim report. British Stomach Cancer Group.

**DOI:** 10.1038/bjc.1989.350

**Published:** 1989-11

**Authors:** W. H. Allum, M. T. Hallissey, L. C. Ward, M. S. Hockey

**Affiliations:** Department of Surgery, Leicester Royal Infirmary, UK.

## Abstract

A prospective, randomised controlled trial of surgery, surgery with adjuvant radiotherapy and surgery with adjuvant chemotherapy (5-fluorouracil, adriamycin and mitomycin C) in operable gastric cancer is described. Four hundred and thirty-six patients were randomly allocated to one of three treatment groups. With 12 months' minimum follow-up, 334 patients have died, 292 from recurrent cancer. The median survival for all patients was 15 months. Neither form of adjuvant therapy provides any survival advantage. Surgery remains the principal treatment for operable gastric cancer. Care should be taken to standardise surgical treatment and any adjuvant treatments must be compared within the confines of controlled, randomised trials.


### A controlled, prospective, randomised trial of adjuvant chemotherapy or radiotherapy in resectable gastric cancer: interim report

W.H. Allum[1], M.T. Hallissey[2], L.C. Ward[3] & M.S. Hockey[4] for the British Stomach Cancer Group*

[1]*Department of Surgery, Clinical Sciences Building, Leicester Royal Infirmary, PO Box 65, Leicester LE2 7LX;* [2]*Department of Surgery and* [3]*WMCRC Clinical Trials Unit, Queen Elizabeth Hospital, Birmingham B15 2TH; and* [4]*Department of Accident and Emergency Medicine, Royal Hallamshire Hospital, Glossop Road, Sheffield, UK.*

**Summary** A prospective, randomised controlled trial of surgery, surgery with adjuvant radiotherapy and surgery with adjuvant chemotherapy (5-fluorouracil, adriamycin and mitomycin C) in operable gastric cancer is described. Four hundred and thirty-six patients were randomly allocated to one of three treatment groups. With 12 months' minimum follow-up, 334 patients have died, 292 from recurrent cancer. The median survival for all patients was 15 months. Neither form of adjuvant therapy provides any survival advantage. Surgery remains the principal treatment for operable gastric cancer. Care should be taken to standardise surgical treatment and any adjuvant treatments must be compared within the confines of controlled, randomised trials.

Despite the reported decline in the incidence and death rate from gastric cancer throughout the world (Day, 1980), it will remain a major clinical problem for the foreseeable future. Surgery has formed the main treatment for gastric cancer and, as in many solid tumours, salvage therapy for unresectable or recurrent disease has failed to influence survival. The Japanese have reported improved results for surgical treatment of gastric cancer (Miwa, 1979), using strict rules for surgery and pathological examination (Japanese Research Society for Gastric Cancer 1981). In the West, however, there has been almost no progress in the management of this condition in recent years, and this has led to a fatalistic attitude to treatment among many clinicians. The work from Japan has demonstrated that progress is possible and has shown the importance of careful documentation and auditing both to establish the best form of surgery and to assess the value of any adjuvant treatment.

The British Stomach Cancer Group (BSCG) designed a prospective, randomised controlled trial to compare surgery alone with surgery and adjuvant radiotherapy or surgery and adjuvant chemotherapy in operable disease. The protocol emphasised detailed recording of surgical procedures together with full documentation and thorough review of the resected specimen in order to stage disease accurately. The chemotherapy regimen comprised 5-fluorouracil (5-FU), adriamycin and mitomycin C, a combination which in studies of advanced disease has produced a 42% response rate (MacDonald *et al.*, 1980) and gave a 37% response rate in a pilot study of the BSCG variant (MAF) (P.F.M. Wrigley; personal communication).

The incorporation of adjuvant radiotherapy was based on evidence from autopsy and re-operation series which has consistently demonstrated that the stomach bed and regional nodes were the most common sites of failure either alone or in combination with distant metastases (McNeer *et al.*, 1951; Gunderson & Sosin, 1982). Furthermore, in selected patients modest doses of megavoltage irradiation has improved survival at 2 years when compared with a control group undergoing gastric resection only (Robinson & Cohen, 1977).

Recruitment to this trial was completed between June 1981 and July 1986. This report describes the details of this trial and evaluates the initial results.

*Committee of the British Stomach Cancer Group and research registrars: S.J. Arnott, V. Brookes, J. Craven, D.J. Ellis, S.L. Fagg, J.W.L. Fielding, K. Kelly, W. Gregory, D. Levison, W.A.F. McAdam, A. Minawa, A.R. Timothy, J.A.H. Waterhouse, H.S. Winsey and P.F.M. Wrigley.
Correspondence: W.H. Allum.
Received 10 March 1989; and in revised form 11 July 1989.

### Materials and methods

A prospective, randomised controlled trial of adjuvant chemotherapy and radiotherapy following gastric resection for adenocarcinoma recruited patients from 10 centres throughout the United Kingdom. Participating centres were the West Midlands, London, Manchester, Airedale, York, Bristol, Swansea, Sunderland, Leeds and Edinburgh.

Patients eligible for entry to the trial were aged between 15 and 74 years and had undergone surgical resection for adenocarcinoma of the stomach. Patients were staged using a clinicopathological system (Table I). All patients entered into the study gave informed consent.

Those cases excluded were stage I, IVAii or IVB disease, those who had previous significant malignant disease or prior cytotoxic or radiation therapy. In addition, patients were excluded from randomisation where there was any intestinal or biliary obstruction (unrelieved by surgery), impaired renal function (blood urea greater than 9 $mmol\,l^{-1}$ or serum creatinine greater than 120 $mmol\,l^{-1}$), or concurrent cardiac failure.

Before randomisation patients were stratified by centre according to age (younger than 60 years or 60 years and over), length of history (less than 6 months and 6 months or longer) and stage of disease.

Randomisation was into one of three treatment groups: surgery alone, surgery and radiotherapy to the tumour bed

**Table I** Staging system used

| *Stage* | *Clinicopathological details* |
|---|---|
| I | Mucosa +ve<br>Submucosa +ve or −ve<br>Muscularis propria +ve or −ve<br>Serosa −ve<br>Nodes −ve |
| II | Mucosa +ve<br>Submucosa +ve<br>Muscularis propria +ve<br>Serosa +ve<br>Nodes −ve |
| III | Mucosa +ve<br>Submucosa +ve or −ve<br>Muscularis propria +ve or −ve<br>Serosa +ve or −ve<br>Nodes +ve |
| IVA | Resectable<br>(i) Local residual disease<br>(ii) Metastatic residual disease |
| IVB | Unresectable<br>(i) Locally advanced<br>(ii) Metastatic |

and surgery and postoperative combination cytotoxic chemotherapy. Randomisation was performed by the West Midlands Regional Cancer Registry. Data collection and analysis were carried out by the West Midlands Cancer Research Campaign Clinical Trials Unit.

Participating surgeons were asked to document the macroscopic extent of the primary tumour and make a detailed assessment of lymph node and other visceral involvement. For patients undergoing curative surgery, an R1 resection was recommended. Total gastrectomy was not routinely advocated for lesions proximal to the antrum. As a result subtotal and proximal partial gastrectomies were included for mid and proximal lesions respectively. Resections were deemed palliative if low volume local disease remained (stage IVai). More extensive, yet resectable lesions (stage IVaii) were excluded. During the procedure, surgeons were requested to mark the splenic hilum, porta hepatis and any gross residual disease with metal clips for subsequent radiotherapy planning.

On pathological examination the resected specimens were measured in either the fixed or unfixed state. Macroscopic and microscopic description of the specimen included assessment of the depth of penetration and the involvement of proximal and distal resection margins. Participating pathologists were asked to dissect lymph nodes from the cardia, lesser curve, pylorus, greater curve, splenic hilum, posterior pancreas, left gastric artery and coeliac axis and to note the number infiltrated at each site. Any involvement of other resected organs was recorded. A central pathology panel reviewed the sections from resected specimens and classified them according to both the World Health Organization and Lauren classifications.

The chemotherapy regimen consisted of mitomycin C $4\,mg\,m^{-2}$, adriamycin $30\,mg\,m^{-2}$ and 5-fluorouracil $600\,mg\,m^{-2}$ given intravenously on one day only (MAF). The first course was to be administered within 4 weeks of surgery and repeated at 3-weekly intervals for a total of eight courses. Dosages were modified according to haematological and biochemical parameters including urinalysis for proteinuria. Half doses were given if the white blood cell count was in the range $2.0-3.0 \times 10^9\,l^{-1}$ or the platelet count was $100-125 \times 10^9\,l^{-1}$. If the counts were below the lower limits of these ranges, no drugs were given and blood counts were checked weekly until adequate levels returned. Proteinuria on two successive occasions or a rise in blood urea to $8\,mmol\,l^{-1}$ or serum creatinine to $150\,mmol\,l^{-1}$ were indications to stop administration of mitomycin C.

Patients randomised to radiotherapy were required to undergo intravenous pyelography, and be planned on either a simulator or a diagnostic X-ray unit. Radiotherapy was administered using AP/PA parallel opposed portals to include the porta hepatis and splenic hilum as marked at surgery. Appropriate renal shielding was employed to exclude as much renal tissue from the treatment field as possible. A mid line tumour dose of 4,500 cGy in 25 fractions over 35 days was given using megavoltage equipment (cobalt-60 or linear accelerator). A further boost dose of 500 cGy to a reduced field could be given at the discretion of the radiotherapist. Blood counts were monitored weekly throughout treatment.

All patients were to be seen every 3 weeks for the first 6 months after surgery. Thereafter follow-up was to be every 6 weeks for 2 years and subsequently at 3-monthly intervals. At each visit data sheets were completed to document clinical progress, including evidence of recurrence. Haematological and biochemical parameters were recorded together with details of any side-effects from treatment. Clinicians were requested to provide post mortem evidence of disease status at death whenever possible.

Survival has been taken as the only criterion of response. In addition to the clinical follow-up within the trial, the completeness of the notification of death was verified by the registration of patients with the West Midlands Regional Cancer Registry. All randomised patients were included in the survival analysis. The survival of live patients was censored at 1 July 1987 when all had complete follow-up. The probability of survival has been estimated by the life-table method (Kaplan & Meier, 1958) and statistical comparisions made with the log rank test (Peto et al., 1977).

### Results

### Patients

During the 5 years of recruitment 436 patients were entered into the study. The data were analysed when there was a minimum follow-up of 12 months. After randomisation there were 145 in the surgery only group, 153 in the radiotherapy group and 138 in the chemotherapy group. The distribution of patient characteristics within each treatment group is shown in Table II.

After completion of recruitment, the eligibility of all patients was assessed according to the protocol criteria for inclusion in the trial. Details of the 25 entry criteria violations found are shown in Table III.

Six patients died within 30 days of operation. Three died of their disease, one of a myocardial infarct and two of complications of surgery. All had already been randomised, to surgery alone (one patient), radiotherapy (two patients) and chemotherapy (three patients).

Randomisation was achieved within a month of operation in 409 (95%) of the 436 patients entered. The median time to randomisation was 13 days with a range of 1–82 days. The time from operation to start of treatment varied with the treatment drawn. The 145 patients in the surgery group were placed on routine review with 49 (34%) being seen in the first month and 66 (46%) being seen in the second. For the 153 patients randomised to radiotherapy 31 (20%) began radiotherapy within 1 month of operation and 60 (39%) within the second. By comparision 74 (54%) of the 138 randomised to chemotherapy were treated in the first month and 37 (27%) in the second. Treatment commenced within a month of operation in 105 (45%) of the 232 cases who received adjuvant therapy.

### Treatment

*Chemotherapy* The number of cycles given to each of the 138 patients randomised to receive chemotherapy is shown in Table IV. Twenty-three (17%) patients did not receive chemotherapy. They either refused (11 patients), were too ill or had died (9 patients) or had pre-existing cardiac disease (2 patients). In one case the reason is not known.

Thirty patients (22%) received less than six cycles of chemotherapy. In this group, progressive ill health (13 patients) and refusal (12 patients) were the major reasons for stopping. The remaining indications were debilitating vomiting and nausea (3 patients), haematological and biochemical toxicity (1 patients) and hypotension (1 patient).

Eight-five (62%) of the 138 patients completed six or more cycles with 58 (42%) completing the planned eight cycles. In the 27 patients having six to seven cycles, seven patients became too unwell and two refused to continue the final cycles. Side-effects halting treatment were haematological and biochemical toxicity (7 patients), debilitating nausea and vomiting (2 patients) and allergy to one of the agents (6 patients). Hypotension caused one patient to stop and in two patients the reason for failure to complete the treatment programme is not known.

The indications for the modification of chemotherapy have already been described. Of the 115 who started treatment, 75 completed their given cycles without dose modification. The commonest reasons for modification were haematological changes (19 patients) and evidence of renal dysfunction (10 patients). The remaining reasons and cycle at which dose modifications were made are shown in Table V.

*Radiotherapy* One hundred and seventeen patients of the 153 randomised to radiotherapy received treatment. Nineteen patients failed to start radiotherapy due to death or poor

**Table II** Patient characteristics by treatment group

| | *Treatment group* | | | | | | | |
|---|---|---|---|---|---|---|---|---|
| | *Surgery (n = 145)* | | *Radiotherapy (n = 153)* | | *MAF (n = 138)* | | *All patients* | |
| *Continuous data* | | | | | | | | |
| Age (years)[a] | 63 | | 65 | | 63 | | 64 | |
| Duration of symptoms (months)[a] | 5 | | 5 | | 5 | | 5 | |
| Weight loss (as % of normal weight)[a] | 10 | | 10 | | 10 | | 10 | |
| *Categorical data* | | | | | | | | |
| Sex | n | % | n | % | n | % | n | % |
| Male | 106 | 73 | 99 | 65 | 98 | 71 | 303 | 69 |
| Female | 39 | 27 | 54 | 35 | 40 | 29 | 133 | 31 |
| Stage | | | | | | | | |
| II | 26 | 18 | 25 | 16 | 22 | 16 | 73 | 17 |
| III | 76 | 52 | 83 | 54 | 76 | 55 | 235 | 54 |
| IVAi | 43 | 30 | 45 | 29 | 40 | 29 | 128 | 29 |
| Serosal involvement | 135 | 93 | 141 | 92 | 120 | 87 | 396 | 91 |
| Node involvement | 110 | 76 | 122 | 80 | 103 | 75 | 335 | 77 |
| Resection line involvement | 23 | 16 | 27 | 18 | 28 | 20 | 78 | 18 |
| Residual disease present | 35 | 24 | 33 | 22 | 25 | 18 | 93 | 21 |
| Resection | | | | | | | | |
| Radical (R1) | 125 | 86 | 129 | 84 | 119 | 86 | 373 | 86 |
| Palliative | 20 | 14 | 24 | 16 | 19 | 14 | 63 | 14 |

[a]Median values.

**Table III** Protocol violations by treatment group

| | *Surgery* | *Radiotherapy* | *Chemotherapy* | *Total* |
|---|---|---|---|---|
| Too old | – | 1 | 1 | 2 |
| Histology[a] | 1(NH lymphoma) | 1(NH lymphoma) | 1(Carcinoid) | 3 |
| Prior malignancy[b] | 1 | 3 | – | 4 |
| Cardiac disease[c] | – | – | 2 | 2 |
| Stage I | – | 1 | 2 | 3 |
| Stage IVAii | 5 | 4 | 2 | 11 |
| Total | 7 | 10 | 8 | 25 |

[a]Not gastric adenocarcinoma on review. [b]One cervix carcinoma, one melanoma, one rectum carcinoma, one bladder papilloma. [c]Not assessable in other groups.

**Table IV** Numbers of patients according to chemotherapy cycles received

| *Number of cycles* | *Number of patients* | *%* |
|---|---|---|
| 0 | 23 | 17 |
| 1 | 6 | 4 |
| 2 | 6 | 4 |
| 3 | 5 | 4 |
| 4 | 10 | 7 |
| 5 | 3 | 2 |
| 6 | 13 | 9 |
| 7 | 14 | 11 |
| 8 | 58 | 42 |
| Total | 138 | 100 |

**Table V** Time of first dose modification by cause

| | *Cycle number* | | | | | | | | |
|---|---|---|---|---|---|---|---|---|---|
| *Cause of modification* | *1* | *2* | *3* | *4* | *5* | *6* | *7* | *8* | *Total* |
| Haematology | – | 4 | 1 | 4 | 2 | 1 | 6 | 1 | 19 |
| Urea | 2 | 2 | 2 | 1 | 1 | – | 2 | – | 10 |
| Allergy | – | – | – | – | 2 | 2 | – | – | 4 |
| Cardiac | 1 | – | – | 3 | – | – | – | – | 4 |
| Vomiting/nausea | – | – | – | – | – | – | – | 1 | 1 |
| Hypotension | – | – | 1 | – | – | – | – | – | 1 |
| Reason not known | – | – | – | – | 1 | – | – | – | 1 |
| Total | 3 | 6 | 4 | 8 | 6 | 3 | 8 | 2 | 40 |

general condition and 13 patients refused to start radiotherapy. Four patients who failed to fulfil the entry criteria (two stage IVAii, one too old, one lymphoma) were unsuitable to start treatment.

The protocol defined dose of 4,500 cGy (± 10%) was administered in 102 patients. Two patients received the additional 500 cGy boost dose to a reduced field. In 13 patients lesser doses were given. One centre elected to vary from the protocol by giving 3,700 cGy (± 10%) in 16 fractions over 21 days (8 patients). Poor condition or progression of disease caused a dose reduction of more than 10% in four patients, and gastrointestinal toxicity required a dose reduction in one patient.

Symptomatic side-effects were recorded for all patients on radiotherapy. In 55 cases no side-effects were seen. Mild nausea and vomiting occurred in 48 patients, and was severe in one further patient. A low white blood count was documented in two patients. Desquamation in the treated area was reported in five cases. Poor tolerance due to poor general condition was seen in six cases.

*Survival*

The median duration of survival was 15 months. Stage had a significant influence on survival (Figure 1), but there was no significant effect for the other stratification variables (age $\chi^2_1 = 0.01$, $P = 0.94$; duration of symptoms $\chi^2_1 = 0.49$, $P = 0.49$; centre $\chi^2_7 = 4.0$, $P = 0.79$).

*Effect of treatment*

The three treatment groups do not differ significantly in their survival (Figure 2; $\chi^2_2 = 5.3$, $P = 0.07$). Neither the chemotherapy group ($\chi^2_1 = 0.8$, $P = 0.36$) nor the radiotherapy group ($\chi^2_1 = 1.8$, $P = 0.18$) differ from the control group.

Details of the site and date of first recurrence have been recorded in 172 cases. These data are largely based on clinical findings made at routine follow up. Further investigations performed in this group included nine second look laparotomies. The site of recurrence has been classified as local to the gastric bed or regional nodes, distant metastasis

| Number of patients At risk | 0 | 12 | 24 | 36 | 48 | 60 | 72 |
|---|---|---|---|---|---|---|---|
| Stage II | 73 | 61 | 47 | 33 | 21 | 17 | – |
| Stage III | 235 | 142 | 78 | 50 | 31 | 19 | – |
| Stage IVAi | 128 | 48 | 14 | 8 | – | – | – |

**Figure 1** Survival by stage (stage II, 39/73 deaths; stage III, 175/235 deaths; stage IVAi, 120/128 deaths; $\chi^2_2$ <0.0001, $P = 72.3$).

| Number of patients At risk | 0 | 12 | 24 | 36 | 48 | 60 | 72 |
|---|---|---|---|---|---|---|---|
| Surgery (S) | 145 | 82 | 45 | 31 | 17 | 13 | – |
| Radiotherapy (R) | 153 | 79 | 43 | 26 | 13 | 9 | – |
| Chemotherapy (M) | 138 | 90 | 48 | 34 | 20 | 18 | – |

**Figure 2** Survival by treatment group (surgery group, 110/145 deaths; radiotherapy group, 123/153 deaths; chemotherapy group, 101/138 deaths; $\chi^2_2 = 5.3$, $P = 0.07$).

**Table VI** Site of first recurrence by treatment group

| | *Surgery* | *Radiotherapy* | *Chemotherapy* | *Total* |
|---|---|---|---|---|
| Local/regional | 37 | 14 | 21 | 72 |
| Distant | 30 | 29 | 33 | 92 |
| Both | 2 | 1 | 5 | 8 |
| Total | 69 | 44 | 59 | 172 |

**Table VII** Cause of death by treatment group

| | *Surgery* | *Radiotherapy* | *Chemotherapy* | *Total* |
|---|---|---|---|---|
| Cancer | 101 | 102 | 89 | 292 |
| Complications | 0 | 2 | 4 | 6 |
| Second primary | 1 | 4 | 2 | 7 |
| Other[a] | 7 | 12 | 5 | 24 |
| Cause unknown | 1 | 3 | 1 | 5 |
| Total | 110 | 123 | 101 | 334 |

[a]Four had recurrent disease present at death.

or both (Table VI). These data, though based on clinical findings, show a lower local and regional relapse rate in those receiving adjuvant treatment ($\chi^2 = 10.7$, $P<0.01$).

*Cause of death*

At the time of analysis 334 (77%) of the patients admitted to the trial had died, 110 in the surgery alone group, 123 in the radiotherapy group and 101 in the chemotherapy group. The cause of death (Table VII) has been obtained from the clinican in charge of the case, general practitioners or from cancer registries. Autopsies were performed on 35 (11%).

Two hundred and ninety-two deaths were due to recurrent stomach cancer. Other causes were responsible for the death of 37 patients. Seven patients died from other primary cancers, of which three were bronchogenic, and one each of melanoma, larynx, prostate and colon. Fourteen deaths were secondary to cardiovascular or cerebrovascular disease. This group includes seven acute myocardial deaths, six of which occurred in the radiotherapy group. General deterioration in health caused 10 deaths. Six patients died as a result of surgical complications, one of which followed a second gastric resection for recurrent disease. The cause of death is not known in five cases.

In summary, 37 patients are known to have died from causes other than gastric cancer, eight of whom were randomised to surgery alone, 18 to radiotherapy and 11 to chemotherapy. This difference in the proportion of non-cancer deaths between the three treatment groups is not statistically significant ($\chi^2 = 3.5$, $0.1 < P < 0.2$).

### Discussion

The study described in this report was set up to evaluate the role of two modalities of adjuvant treatment in advanced gastric cancinoma. The trial was designed to incorporate the detailed documentation recommended by Japanese workers and was extended to include a central pathology review. A detailed record of the surgical procedures undertaken in each patient was made and all histological specimens obtained at operation were subjected to thorough review to ensure consistent pathological grading and staging. The adjuvant treatments assessed were short-term chemotherapy using the most effective agents in combination and local radiotherapy to the tumour bed. In this interim report the results of recruitment, randomisation and the therapeutic regimens have been presented. The clinicopathological data will form the subject of a future report.

The cytotoxic agents evaluated in this study have been reported to have produced the best response rates seen in the treatment of advanced or recurrent gastric cancer (Earl *et al.*, 1984). There is no doubt that response rates in advanced disease are important indicators of sensitivity to cytoxic agents. However, careful review of the reports of these studies demonstrate very few complete and durable responses. Indeed one of the larger studies, using 5-FU, adriamycin and mitomycin C, documents only two complete responses lasting for 23 and 29 months respectively (Cunningham *et al.*, 1984).

The results of this study demonstrate that partial response rates produced by these agents fail to translate into a survival advantage in an adjuvant setting. In a report of a similar study evaluating the same three cytotoxic agents, but at different doses, the preliminary results demonstrate a modest early survival advantage for the treated group (Schein *et al.*, 1986). It remains to be seen whether this advantage will persist.

There have been reports of complete responses to radiotherapy given as palliation for advanced disease (Gunderson & O'Connell, 1984; Weiland & Hymmen, 1970), though these are rare. The difficulties in the use of radiotherapy in the treatment of gastric cancer stem from the sensitivity of the surrounding structures which limit the dose that can be used (Gunderson, 1986). Studies have demonstrated that the sites of local failure can be encompassed in a conventional radiotherapy field (Gunderson & Sosin, 1982). Using such a field, the interim results of this study demonstrate no influence on survival from the use of adjuvant radiotherapy.

Adjuvant therapy aimed at treating microscopic or macroscopic disease may theoretically result in prolongation of survival. It is evident that eradication of large volumes of modestly sensitive tumours by cytotoxics may now be considered optimistic if not naive. Models of tumour growth indicate that tumours are most rapidly dividing when small in volume (Carter *et al.*, 1988) and most cytotoxic agents act optimally on the dividing cells. The timing of adjuvant therapy in those with microscopic disease may be crucial. This has been demonstrated in breast cancer (Nissen-Meyer *et al.*, 1978) and gastric cancer (Imanaga & Nakazato, 1977; Nakajima *et al.*, 1978). Treatment in the perioperative period when tumour burden is minimal may provide the means to improve on results with currently available agents.

Large multicentre studies such as the one reported here and its predecessor (Allum *et al.*, 1989) demonstrate how groups of clinicans can execute complex chemotherapy schedules and also undertake close review of the effects of such treatment. However, such studies equally demonstrate the difficult logistics of adherence to protocols. The first trial had demonstrated a significant improvement in survival at 1 year for those treated in the first month (Fielding *et al.*, 1983). The aim in this second study was to commence therapy within a month of surgery. This was achieved in only 45% of patients who received adjuvant treatment. This must in part reflect the scheduling of radiotherapy machine time and the variable postoperative recovery of patients.

The other major problem encountered in this study was inability to complete the treatment schedules. Only 62% of those randomised to chemotherapy completed six or more of the recommended eight cycles. The major reasons for failure to complete chemotherapy were early death and progressive disease with toxicity having a minor role. In the radiotherapy group, there was closer adherence to the recommended treatment, with 104 of the 117 who started treatment receiving the correct dose. Little significant toxicity was seen in the radiotherapy group, with dose reduction due to toxicity in only one patient. However, a large number (24%) failed to start radiotherapy as randomised. Again, ill health and early death prevented patients from starting treatment. No patient in this trial suffered severe, life threatening toxicity as a result of either adjuvant treatment.

Deviations from the protocol become more important when the effect of treatment is considered. In addition the difficulty of establishing the precise site and time of recurrence has led to survival being taken as the only determinant of response in this study. However, the clinical information that is available for a number of patients suggests that the distribution of recurrence may be altered by the adjuvant modalities. Local control was apparently improved in those treated by irradiation or chemotherapy; however, as the site of recurrence cannot be evaluated for all patients stated to have died with disease, these data must be viewed with caution.

The results of the present study have failed to provide any evidence that the adjuvant therapies evaluated influence survival. The use of adjuvant treatment in this disease should be restricted to randomised, controlled trials where their effect can be properly assessed.

In gastric cancer, surgery at an early stage remains the goal. Unfortunately most patients in the United Kingdom currently present with disease which is not confined to the stomach. Standardisation of surgical precedure and documentation of operative and pathological findings are of paramount importance in order to compare the results of adjuvant regimens. This second trial attempted to address this problem in an open, multicentre setting. The Medical Research Council are currently undertaking a randomised trial to evaluate surgical techniques in cases with curable disease. It is only by such critical evaluation of treatment that progress is likely in this disease and in the other common solid tumours in which little improvement in prognosis has been seen over the past 20 years.

This work was supported by a grant from the Cancer Research Campaign.

The British Stomach Cancer Group and consultants contributing patients:
B.C. Abernathy, Victoria Hospital, Kircaldy
J. Alexander-Williams, General Hospital, Birmingham
P.R. Armistead, General Hospital, Kidderminster
D.V. Ash, Cookridge Hospital, Leeds
D.J. Ashley, Morriston Hospital, Swansea
F. Ashton, Queen Elizabeth Hospital, Birmingham
R.S. Atkinson, Newcastle General Hospital, Newcastle on Tyne
R.M. Baddeley, General Hospital, Birmingham
E.T Bainbridge, Sandwell District General Hospital, West Bromwich
J. Bancewicz, Hope Hospital, Salford
A. Banks, Queen Elizabeth Hospital, Birmingham
G.H. Berry, Cookridge Hospital, Leeds
A.J. Blackshaw, Bedford General Hospital
N.L. Browse, St Thomas' Hospital, London
J.M. Buchanan, North Staffordshire Royal Infirmary, Stoke on Trent
R. Buchan, Victoria Hospital, Kircaldy
J.A. Bullimore, Bristol Royal Infirmary, Bristol
J.H. Burman, Bromsgrove General Hospital, Bromsgrove

K. Burnand, St Thomas' Hospital, London
D. Cade, Leighton Hospital, Crewe
D.J. Campbell, Selly Oak Hospital, Birmingham
L.J. Chalstrey, St Bartholemews Hospital, London
A.D. Chetiyawardana, Queen Elizabeth Hospital, Birmingham
J. Cook, Eastern General Hospital, Edinburgh
J.R.N. Curt, Salford Royal Hospital, Salford
S.A. Davies, North Middlesex Hospital, London
D. Day, Torbay Hospital, Torquay
H.C. De Castella, Burton General Hospital, Burton on Trent
J.M. Dolphin, The Manor Hospital, Walsall
N.J. Dorricott, General Hospital, Birmingham
H.A.F. Dudley, St Marys Hospital, London
J.S. Duthie, Oldham and District General Hospital, Oldham
D.J. Fairlamb, New Cross Hospital, Wolverhampton
K.D. Fortes-Mayer, The Manor Hospital, Walsall
J.F. Forrest, North Staffordshire Royal Infirmary, Stoke on Trent
K.M. Fussell, Wigan Infirmary, Wigan
G.R. Giles, St James University Hospital, Leeds
E.W. Gillison, General Hospital, Kidderminster
S. Glick, Burton General Hospital, Burton on Trent
M.D. Goldman, East Birmingham Hospital, Birmingham
J.P. Grant, Selly Oak Hospital, Birmingham
G.F. Grave, Alexandria Hospital, Reddich
R. Grieve, Walsgrave Hospital, Coventry
R.P. Grimley, Wordsley Hospital, Stourbridge
R. Hall, York District Hospital, York
J.D. Hamer, Queen Elizabeth Hospital, Birmingham
D.M. Hancock, Sunderland District General Hospital, Sunderland
J.D. Hennessy, Sandwell District General Hospital, West Bromwich
G.M. Hoare, Altringham General Hospital, Altringham
M. Hoyle, Special Projects Coordinator, Airedale General Hospital, Keighley
J.M. Howat, North Manchester General Hospital, Manchester
W.V. Humphreys, Oldham Royal Infirmary, Oldham
M.H. Irving, Hope Hospital, Salford
B.T. Jackson, St Thomas' Hospital, London
A. Jewkes, Queen Elizabeth Hospital, Birmingham
B.G. Jones, Queen Elizabeth Hospital, Birmingham
M.R.B. Keighley, General Hospital, Birmingham
S.C. Kennedy, East Birmingham Hospital, Birmingham
R.D. Kingston, Park Hospital, Manchester
J.H. Kirkham, Sandwell District General Hospital, West Bromwich
T.N. Latief, Queen Elizabeth Hospital, Birmingham
D.J. Leaper, Bristol Royal Infirmary, Bristol
S. Leveson, St James University Hospital, Leeds
W.M. Lien, Good Hope General Hospital, Sutton Coldfield
G. Little, The Manor Hospital, Walsall
J. Low (Research Nurse), Queen Elizabeth Hospital, Birmingham
W. Maley (Research Nurse), Park Hospital, Manchester
M.C. Mason, Singleton Hospital, Swansea
N.K. Maybury, Wigan Infirmary, Wigan
P. McMaster, Queen Elizabeth Hospital, Birmingham
M.D. Middleton, East Birmingham Hospital, Birmingham
N. Morteson, Bristol Royal Infirmary, Bristol
H. Norcott, Corbett Hospital, Stourbridge
G.D. Oates, General Hospital, Birmingham
M.L. Obeid, Dudley Road Hospital, Birmingham
R.W. Parker, Walsgrave Hospital, Coventry
R. Payne, North Middlesex Hospital, London
J.H. Peacock, Bristol Royal Infirmary, Bristol
J. Powell, West Midlands Regional Cancer Registry, Birmingham
T. Priestman, Queen Elizabeth Hospital, Birmingham
A. Rhodes, Walsgrave Hospital, Coventry
R.S. Rihan, Good Hope General Hospital, Sutton Coldfield
R.H. Sage, Selly Oak Hospital, Birmingham
J.H. Scarffe, Christie Hospital and Holt Radium Institute, Manchester
J. Scoble, Northstaffordshire Royal Infirmary, Stoke on Trent
W.S. Shand, St Bartholemews Hospital, London
M. Simms, Selly Oak Hospital, Birmingham
A. Simpson, Sandwell District General Hospital, West Bromwich
G. Slaney, Queen Elizabeth Hospital, Birmingham
G.S. Sokhi, East Birmingham Hospital, Birmingham
J.L. Somervell, The Manor Hospital, Walsall
D. Spooner, Queen Elizabeth Hospital, Birmingham
R.D. Stedeford, Oldchurch Hospital, Romford
R.A. Stockley, General Hospital, Birmingham
J.G. Temple, Queen Elizabeth Hospital, Birmingham
D.R. Thomas, Good Hope General Hospital, Sutton Coldfield
R.W. Tudor, Solihull Hospital, Solihull
D.E.F. Tweedle, Withington Hospital, Manchester
S.G. Vaidya, Oldchurch Hospital, Romford
T.A. Waterworth, St Cross Hospital, Rugby
D.C.T. Watson, East Birmingham Hospital, Birmingham
M.C. White, Gulson Hospital, Coventry
N.E. Winston, Selly Oak Hospital, Birmingham
R.J. Williams, North Manchester General Hospital, Manchester
D.A.K. Woodward, Walsgrave Hospital, Coventry
R.C.N. Williamson, Bristol Royal Infirmary, Bristol
P.L.C. Xavier, Oldchurch Hospital, Romford
A.E. Young, St Thomas' Hospital, London

### References

ALLUM, W.H., HALLISSEY, M.T. & KELLY, K.A. (1989). Adjuvant chemotherapy in operable gastric cancer – 5 year follow-up of first British Stomach Cancer Group Trial. *Lancet*, **i**, 571.

CARTER, S.K., BAKOWSKI, H.T. & HELLMAN, K. (1988). *Chemotherapy of Cancer*. Churchill Livingstone: New York.

CUNNINGHAM, D., SOUKOP, M., McCARDLE, C.S. & 8 others (1984). Advanced gastric cancer: experience in Scotland using 5-fluorouracil and mitomycin C. *Br. J. Surg.*, **71**, 673.

DAY, D.W. (1980). Epidemiology and pathology of gastric cancer. In *Recent Advances in Gastrointestinal Pathology*, Wright, R. (ed) p. 285. W.B. Saunders: Philadelphia.

EARL, H.M., COOMBES, R.C. & SCHEIN, P.S. (1984). Cytotoxic chemotherapy for cancer of the stomach. *Clin. Oncol.*, **3**, 351.

FIELDING, J.W.L., FAGG, S.L., JONES, B.G. & 9 others (1983). An interim report of a prospective, randomised controlled study of adjuvant chemotherapy in operable gastric cancer: British Stomach Cancer Group. *World J. Surg.*, **7**, 390.

GUNDERSON, L.L. (1986). Therapeutic options: radiotherapy. In *Gastrointestinal Oncology*, Fielding, J.W.L. & Priestman, T.J. (eds) p. 17. Castle House: Tunbridge Wells.

GUNDERSON, L.L. & O'CONNELL, M.J. (1984). Radiation with and without chemotherapy in gastric cancer. *Clin. Oncol.*, **3**, 327.

GUNDERSON, L.L. & SOSIN, H. (1982). Adenocarcinoma of the stomach: areas of failure in a reoperation series (second or symptomatic looks): clinicopathologic correlation and implications for adjuvant therapy. *Int. J. Radiat. Oncol. Biol. Phys.*, **8**, 1.

IMANAGA, H. & NAKAZATO, H. (1977). Results of surgery for gastric cancer and effect of adjuvant mitomycin C on cancer recurrence. *World J. Surg.*, **1**, 213.

JAPANESE RESEARCH SOCIETY FOR GASTRIC CANCER (1981). The general rules for the gastric cancer study in surgery and pathology. *Jpn. J. Surg.*, **11**, 127.

KAPLAN, E. & MEIER, P. (1958). Non parametric estimation from incomplete observations. *J. Am. Stat. Assoc.*, **53**, 457.

MacDONALD, J.S. SCHEIN, P.S., WOOLLEY, P.V. & 8 others (1980). 5-Fluorouracil, doxorubicin and mitomycin C (FAM) combination chemotherapy for advanced gastric cancer. *Ann. Intern. Med.*, **93**, 533.

McNEER, G., VANDENBERG, H., DONN, F.Y. & BOWDEN, L.A. (1951). A critical evaluation of subtotal gastrectomy for the cure of cancer of the stomach. *Ann. Surg.*, **134**, 2.

MIWA, K. (1979). Cancer of the stomach in Japan. *Gann Monogr. Cancer Res.*, **22**, 61.

NAKAJIMA, T., FUKAMI, A., OHASHI, I. & KAJITANI, T. (1978). Long term follow up study of gastric cancer patients treated with surgery and adjuvant chemotherapy with mitomycin C. *Int. J. Clin. Pharmacol. Biopharm.*, **16**, 209.

NISSEN-MEYER, R., KJELLGREN, K., MALMIO, K., MANSSON, B. & NORIN, T. (1978). Results with one short course of cyclophosphamide after mastectomy for breast cancer. *Cancer*, **41**, 2088.

PETO, R., PIKE, M.C., ARMITAGE, P. & 7 others (1977). Design and analysis of randomised clinical trials requiring prolonged observation of each patient. II Analysis and examples. *Br. J. Cancer*, **35**, 1

ROBINSON, E. & COHEN, Y. (1977). The combination of surgery, radiotherapy and chemotherapy in the treatment of gastric cancer. *Rec. Results Cancer Res.*, **32**, 177.

SCHEIN, P.S., COOMBES, R.C. & CHILVERS, C. (1986). A controlled trial of FAM (5-FU, doxorubicin and mitomycin C) chemotherapy as adjuvant treatment for resected gastric carcinoma: an interim report. *Proc. Am. Soc. Clin. Oncol.*, **5**, 79.

WEILAND, C. & HYMMEN, U. (1970). Megavoltage therapy for malignant gastric tumours. *Strahlentherapie*, **40**, 20.